# Sophorolipids Production by *Candida bombicola* ATCC 22214 and its Potential Application in Microbial Enhanced Oil Recovery

**DOI:** 10.3389/fmicb.2015.01324

**Published:** 2015-11-26

**Authors:** Abdulkadir E. Elshafie, Sanket J. Joshi, Yahya M. Al-Wahaibi, Ali S. Al-Bemani, Saif N. Al-Bahry, Dua’a Al-Maqbali, Ibrahim M. Banat

**Affiliations:** ^1^Department of Biology, College of Science, Sultan Qaboos UniversityMuscat, Oman; ^2^Central Analytical and Applied Research Unit, College of Science, Sultan Qaboos UniversityMuscat, Oman; ^3^Department of Petroleum and Chemical Engineering, College of Engineering, Sultan Qaboos UniversityMuscat, Oman; ^4^School of Biomedical Sciences, University of UlsterColeraine, UK

**Keywords:** biosurfactant, sophorolipids, *Candida bombicola*, microbial enhanced oil recovery, core-flood experiment

## Abstract

Biosurfactant production using *Candida bombicola* ATCC 22214, its characterization and potential applications in enhancing oil recovery were studied at laboratory scale. The seed media and the production media were standardized for optimal growth and biosurfactant production. The production media were tested with different carbon sources: glucose (2%w/v) and corn oil (10%v/v) added separately or concurrently. The samples were collected at 24 h interval up to 120 h and checked for growth (OD_660_), and biosurfactant production [surface tension (ST) and interfacial tension (IFT)]. The medium with both glucose and corn oil gave better biosurfactant production and reduced both ST and IFT to 28.56 + 0.42mN/m and 2.13 + 0.09mN/m, respectively within 72 h. The produced biosurfactant was quite stable at 13–15% salinity, pH range of 2–12, and at temperature up to 100°C. It also produced stable emulsions (%E_24_) with different hydrocarbons (pentane, hexane, heptane, tridecane, tetradecane, hexadecane, 1-methylnaphthalene, 2,2,4,4,6,8-heptamethylnonane, light and heavy crude oil). The produced biosurfactant was extracted using ethyl acetate and characterized as a mixture of sophorolipids (SPLs). The potential of SPLs in enhancing oil recovery was tested using core-flooding experiments under reservoir conditions, where additional 27.27% of residual oil (S_or_) was recovered. This confirmed the potential of SPLs for applications in microbial enhanced oil recovery.

## Introduction

Surfactants are amphiphilic molecules consisting of both hydrophilic and hydrophobic domains that partition preferentially at the interface between fluid phase with different degree of polarity and hydrogen bonding (such as oil and water or air and water). Surfactants intercede in nearly every product and aspect of day to day life, with a total world production exceeding 13 million tons per year (Levison, 2009). Nearly half is used in household and laundry detergents and inevitably ends up in the environment after use, which results in a negative impact on aquatic system since traditional surfactants have low rate of biodegradation, high-ecotoxication and bio-accumulation (Mann and Boddy, 2000; Mann and Bidwell, 2001). Biosurfactants in comparison are a heterogeneous group of surface active molecules synthesized by microorganisms which reduces surface tension (ST) and interfacial tension (IFT) and plays a vital role in microbiological physiology (Al-Sulaimani et al., 2011a). Biosurfactants can be produced from renewable feedstock or even agro-industrial waste products (Joshi et al., 2008; Van Bogaert et al., 2011; Al-Bahry et al., 2013). Biosurfactants have advantages over chemical surfactants, because of their lower toxicity, higher biodegradability, better environmental compatibility and higher selectivity and specific activity at extreme temperature, pH and salinity (Marchant and Banat, 2012a). Biosurfactants have been applied in many fields including the oil industry, both for petroleum production and for incorporation into oil formulations which are considered as the largest possible market (Marchant and Banat, 2012b). Other application related to the oil industries includes oil spill bioremediation/dispersion, both inland and at sea, bioremediation of non-aqueous phase liquids (NAPL), removal/mobilization of oil sludge from storage tanks and to enhance oil recovery (Banat et al., 1991; Sen, 2008; Joshi and Desai, 2010). The second largest market for biosurfactants are emulsion polymerization for paints, paper coating and industrial coatings.

Major classes of biosurfactants are glycolipids, lipopeptides, lipoproteins, phospholipids, fatty acids, polymeric surfactants and particulate surfactants (Desai and Banat, 1997). Glycolipidic biosurfactants are extracellular surface active molecules produced by many microorganisms, e.g., sophorolipids (SPLs) produced by yeast – *Candida bombicola* (formerly called *Torulopsis bombicola*). *C. bombicola* was isolated from bumblebee honey (Kachholz and Schlingmann, 1987). SPLs are amongst one of the most promising biosurfactants. They are produced by non-pathogenic yeast strains; in contrast to rhamnolipids which represent another commercially available glycolipid surfactant that is available industrially, but produced by the opportunistic pathogenic bacteria *Pseudomonas aeruginosa* (Van Bogaert et al., 2007). Generally SPLs are produced as a mixture of slightly different molecules with variations in acetylation and lactonisation. In general, lactonic SPLs are reported to be having better ST lowering and antimicrobial activity, whereas the acidic SPLs are generally better foaming and solubility agents. Furthermore, the acetyl groups render the molecule less water soluble, but enhance their anti-viral and cytokine stimulating effects (Shah et al., 2005). Van Bogaert et al. (2007) reported the production and applications of SPLs produced by various yeast strains.

In this paper we investigated the effect of carbohydrate and oil based media on biosurfactant production by *C. bombicola* ATCC 22214. The biosurfactant was extracted and characterized using high-performance thin layer chromatography – mass spectrometry (HPTLC-MS), matrix-assisted laser desorption ionization – time of flight-mass spectroscopy (MALDI-TOF-MS) and nuclear magnetic resonance (NMR) spectroscopy (^1^H and ^13^C NMR). The stability of the biosurfactant under extreme conditions of pH, temperature and salinity was also investigated and finally we tested its efficacy in enhancing oil recovery using core-flood experiments.

## Materials and Methods

All of the experimental data were expressed in terms of arithmetic averages obtained from at least three independent replicates, with standard deviation (±).

### Chemical and Reagents

All chemicals and reagents used were of analytical grade, media constituents for microbial studies were of microbiology grade, and the hydrocarbons had minimum of 99% purity. All chemicals, reagents and hydrocarbons were purchased from Sigma–Aldrich Co. LLC. Corn oil was purchased from local market. Light and heavy crude oil were kindly provided by local oil company, Petroleum Development Oman (PDO).

### Microorganism and its Maintenance

The yeast *C. bombicola* ATCC22214 was sub- cultured from the stock culture on freshly prepared potato dextrose agar slants (PDA), composition (g/l): potato extract, 4.0; dextrose, 20.0; agar, 15.0) and glucose yeast peptone agar slants (GYPA), composition (g/l): glucose, 100; yeast extract, 30; peptone, 50; agar, 20; and incubated at (25°C) for 48 h. The agar slants were preserved at 5°C.

### Seed Culture Preparation

Two types of seed media were used for developing the seed culture: potato dextrose broth (PDB) and glucose-yeast extract-urea broth (GYUB), composition (g/l): glucose, 50; yeast extract, 5; urea, 0.5. Seed media were prepared and distributed 50 ml each in 250 ml Erlenmeyer flasks and autoclaved at 121°C and 15 psi for 15 min. Seed media flasks were inoculated with a loop full of the microorganism freshly grown on GYPA or PDA agar slant, and incubated for 48 h at 25°C; 160 rpm in a rotary incubator shaker.

### Preparation of Production Media

Three different production media were tested containing (g/l): yeast extract, 10; urea, 1; and different carbon sources. The pH was adjusted to 4.0 using 1M HCl, before autoclaving. Glucose and/or corn oil was used as carbon sources, as: only glucose (2% w/v), only corn oil (10% v/v) or both glucose (2% w/v) and corn oil (10% v/v). Glucose and corn oil were autoclaved separately and added to pre-sterilized production media. The flasks were inoculated with 2% (v/v) pre-grown seed media (GYUB) and incubated in a rotary shaker at 25°C, 160 rpm for 120 h. Samples were withdrawn every 24 h for different analysis: growth (OD_660_), pH, ST, and IFT.

### Surface Tension and Interfacial Tension Measurements

A pendent drop tensiometer (DSA100, KRUSS, Germany) was used to measure ST and IFT. The IFT was measured against hexadecane as the embedding phase as reported by Al-Sulaimani et al. (2011b). All measurements were done in triplicate at ambient temperature (25 ± 2°C) and pressure (1 atm) and the average of three readings were reported for each of the three independent experiments.

### Stability Studies

The stability of biosurfactant produced by *C. bombicola* was studied under various environmental parameters including wide range of temperature, pH and different salt (NaCl) concentrations. The study was carried out by changing the levels of one parameter while keeping other parameters constant using cell-free biosurfactant broth centrifuged at 10,000 × *g* for 15 min. ST and IFT were measured after each treatment to test their effect on biosurfactant activity.

#### Temperature Stability Tests

For the temperature stability test, 25 ml of the cell-free broth were added in 50 ml glass tubes and tightly closed with stopper to prevent evaporation. The tubes were incubated at different temperatures (40, 60, 80, and 100°C) each for 30 min, cooled down to room temperature and the biosurfactant activity was measured.

#### Salinity Stability Tests

Different concentrations of salt (NaCl) were added (1, 3, 5, 7, 10, 13, 15, 20, and 25% w/v) into 25 ml cell-free broth, dissolved completely and the effect on biosurfactant activity was measured.

#### pH Stability Tests

For the pH stability study, 25 ml of cell-free broth was adjusted using either 1N HCl or 1N NaOH to desired values (2, 4, 6, 8, 10, and 12) and the effect on biosurfactant activity was measured.

### Emulsification Index (%E_24_)

The emulsification index (%E_24_) was analyzed using previously reported procedures (Al-Wahaibi et al., 2014; Ismail and Dadrasnia, 2015): two ml of the cell-free broth was added to an equal amount of different hydrocarbons (*n*-hexadecane, heptane, hexane, *n*-tetradecane, 1-methylnaphthalene, *n*-pentane, *n*-tridecane, 2,2,4,4,6,8-heptamethylnonane, heavy crude oil and light crude oil). The solution was mixed by vortexing for 2–3 min at high speed and was left to stand for 24 h. After 24 h the height of the emulsion was measured and the emulsification index was given as the percentage of the height of the emulsified layer divided by the total height of the liquid column multiplied by 100.

### Extraction of Biosurfactant

Biosurfactant was extracted and partially purified by solvent-extraction. The cells were removed by centrifugation (10,000 × *g*, 15 min at 20°C) to obtain cell-free broth. The cell-free broth (1L) was extracted twice with an equal volume of ethyl acetate, shaken vigorously in a separation funnel. The bottom aqueous layer and the top ethyl acetate layers were collected separately. The aqueous portion was re-extracted further twice with ethyl acetate. Ethyl acetate extracts were combined and the solvent was evaporated under vacuum using Rotavapor (Buchi, Switzerland) to give crude biosurfactant along with residual oil. The residual oil was removed by washing thrice with *n*-hexane. Crude biosurfactant was recovered by evaporating the *n*-hexane (Wadekar et al., 2012).

### Biosurfactant Characterization

#### High-Performance Thin Layer Chromatography-Mass Spectroscopy

Biosurfactant was separated and analyzed using an automated HPTLC system (CAMAG, Switzerland) in the Central Analytical and Applied Research Unit (CAARU), Sultan Qaboos University, Oman. Twenty five microliter samples were spotted onto a 10 cm × 10 cm pre-coated silica gel HPTLC plate (Merck, Germany) containing green fluorescent F_254_. These samples were spotted under a flow of nitrogen gas using automatic TLC sampler 4 (ATS 4) spotting device (CAMAG, Switzerland). The plates were developed using an automatic developing chamber ADC 2 (CAMAG, Switzerland) with remote operation from winCATS software, containing solvent systems – MP1: Chloroform: Methanol: Acetic acid (95:5:5) and MP2: Chloroform: Methanol: Water (65:15:2). The documentation and evaluation of the TLC plate was done using TLC visualizer (CAMAG) under direct UV 254 and UV 366 nm light, capturing the images. The separated bands were extracted and eluted by TLC-MS interface (CAMAG, Switzerland), based on the coordinates determined by the TLC scanner 4. The TLC-MS interface head (oval, 4 × 2 mm) was connected to the pump (11 PLUS, HARVARD APPARATUS, Holliston, MA, USA) and the extraction was performed at a flow rate of 10 μl/min, with methanol: acetonitrile (50% diluted with water) – 1:10. The interface outlet was directly connected with the ESI – MS (Qaattro UltimaTM Pt, Micromass^®^, UK), using Mass Lynx V4.0 software. The experimental conditions were: capillary voltage, 3.0 kV; cone voltage, 35 V; lens voltage, 0.0 V; source block temperature, 100°C; desolvation temperature, 120°C; analyzed under both positive and negative modes.

#### Matrix Assisted Laser Desorption Ionization –Time of Flight

All MALDI-TOF experiments were performed at CAARU, Sultan Qaboos University, on UltraFlextreme (Bruker Daltonics, Bremen, Germany) operating in positive reflectron mode in the m/z range of 50–2000 Da. Stainless steel MTP 384 target plate was used for all the molecular weight analysis. Dihydroxy benzoic acid (DHB) dried droplet protocol described in Bruker manual was employed for sample preparation and spotting. Two micro liter of 2, 5-DHB matrix (20 mg/ml) in TA 30 (30:70 v/v ACN:TFA 0.1%TFA) was premixed with 2 μl of the sample solution. One micro liter of the mixture was applied to the ground steel target plate, dried at room temperature. The spectra were acquired using FlexControl software v3.3 (Bruker Daltonics, Bremen, Germany). A SmartBeam-II laser, set at a frequency of 1000 Hz, was used for ionization. The laser strength was optimized at 25–35%. A summed spectrum was obtained for each MALDI-spot. Peaks were detected using the SNAP peak detection algorithm and a baseline subtraction was carried out using “TopHat” algorithm. The MALDI-TOF spectra were externally calibrated using a commercially available peptide mix (peptide calibration standard II, Part-No #222570, Bruker Daltonics, Germany). FlexAnalysis Software v3.3 (Bruker Daltonics) was used for visualization and initial data processing.

#### Nuclear Magnetic Resonance spectroscopy

The NMR experiments were carried out in Bruker Avance III HD 700 MHz spectrometer equipped with 5 mm TCI H/C/N cryoprobe. The proton (^1^H) NMR experiment was run using zg30 pulse program operating at 176.08 MHz. Acquisition parameters were as follows: 90° proton pulse width of 8.00 μs, relaxation delay of 1 s, 16 scans. The proton decoupled ^13^C NMR experiments were carried out using composite pulse decoupling scheme operating at 176.08 MHz. Acquisition parameters were as follows: 90° proton pulse width of 8.00 μs, relaxation delay of 2 s, 512 scans. The Spectra were recorded in CDCl_3_ at 298K and processed using TOPSPIN 3.2 software.

### Core-flooding Experiments

Berea sandstone cores (1.5 inch diameter × 3 inch long) were used for core-flooding experiments. The average porosity and permeability of Berea core-plugs were 20% and 250–350 mD respectively. The formation water and crude oil used in these experiments were obtained from an Omani oil field of interest which has an average reservoir temperature of 60°C. The salinity of the formation water was between 7 and 9% its chemical composition was (kg/m^3^): Sodium, 25.083; Calcium, 3.762; Magnesium, 0.878; Iron, 0.045; Chloride, 47.722; Sulfate, 0.247; Bicarbonate, 0.079. Formation water was filtered prior to use, by Millipore Membrane Filtration Unit (0.45 μm). The crude oil used for core-flood experiments was of API 36.51°. For all core-flooding experiments, cleaned Berea cores were saturated with filtered formation brine using vacuum desiccators for 24 h and pore volume (PV) was determined using the dry and wet weights of the cores. The cores were then flooded with crude oil at 24 cm^3^/h until no more water was produced. The oil initially in place (OIIP) was determined, which was indicated by the volume of water displaced. The cores were subjected to water-flooding at 24 cm^3^/h until no further oil was produced. The residual oil was calculated by measuring the amount of oil produced from the water-flood. Then, 5 PV of the cell-free supernatant (biosurfactant broth) was injected as a tertiary recovery stage and extra oil recovery was determined (Al-Sulaimani et al., 2011b). All core-flood experiments were carried out at 60°C to mimic the average reservoir temperature of the field of interest.

## Results and Discussion

### Biosurfactant Production Studies

**FIGURE 1:**
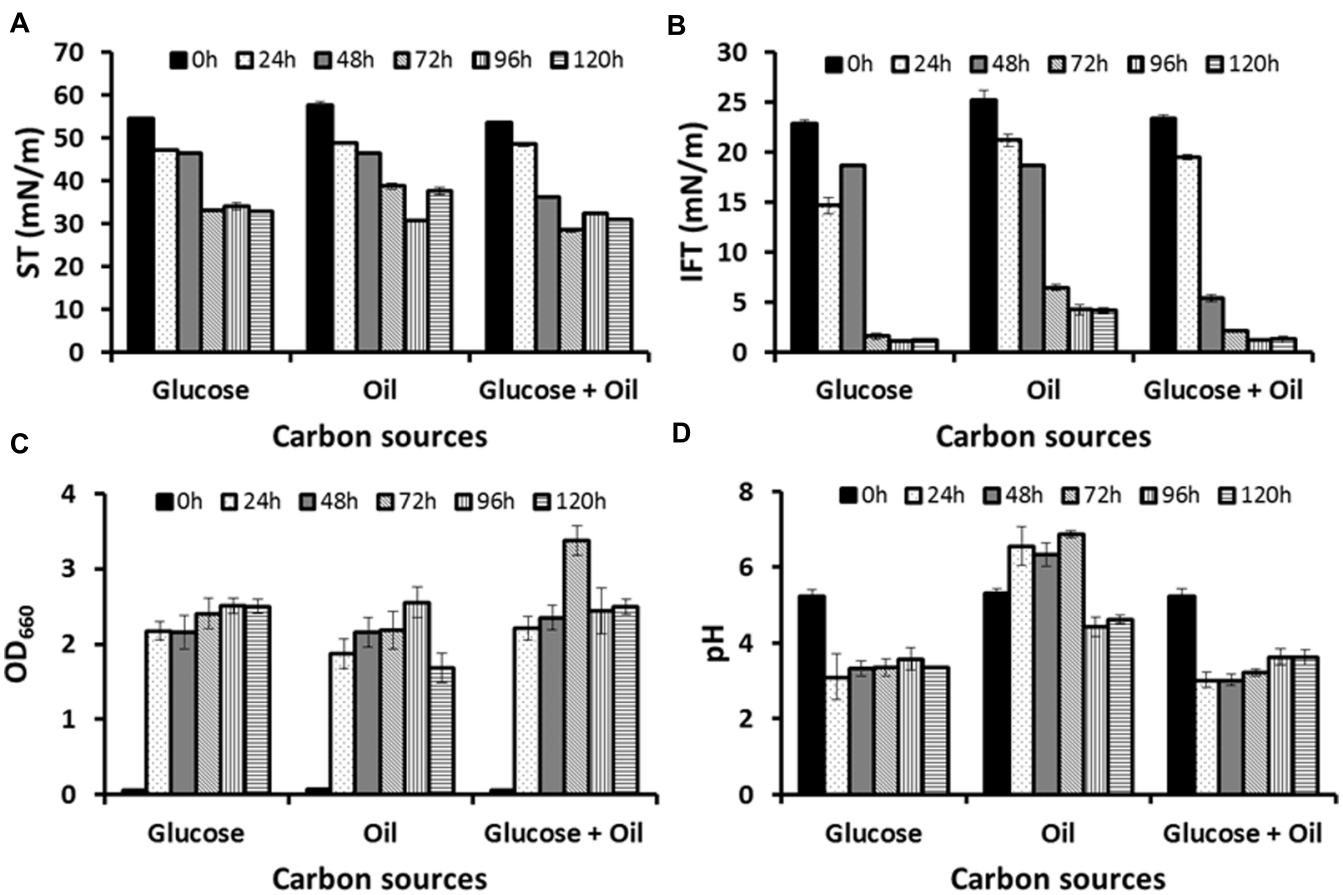
**Production profile for *Candida bombicola* ATCC 22214, when grown in medium with glucose, corn oil or both glucose and corn oil as a carbon source: **(A)** surface tension (ST), **(B)** interfacial tension (IFT), **(C)** Growth – OD_660_, and **(D)** pH profile**.

Two different media GYUB and PDB were tested as seed media of which GYUB supported better growth (GYUB: OD_660_ = 2.441 ±0.02, and PDB: OD_660_ = 2.085 ±0.03) and hence selected as a seed medium for inoculum preparation. To study the biosurfactant production, two different carbon sources (glucose and corn oil) were tested either alone or as a mixture of both. Growth (OD_660_) and biosurfactant production (ST and IFT) were studied from all three different production media up to 120 h. In glucose-based production medium both ST and IFT decreased after 72 h, and reached to 33.08 ±0.05 mN/m and 1.63 ±0.29 mN/m from 54.66 ±0.26 mN/m and 22.84 ±0.36 mN/m respectively, and remained almost stable up to 120 h (**Figures 1A,B**). Maximum growth was observed at 96 h (OD_660_ = 2.508) and didn’t increase further till 120 h (**Figure 1C**). However no change in pH was observed until 120 h (**Figure 1D**). In corn oil-based production medium maximum reduction in ST and IFT was observed after 96 h, and reached to 30.80 ±0.19 mN/m and 4.26 ±0.54 mN/m from 57.81 ±0.79 mN/m and 25.26 ±0.94 mN/m respectively, and remained almost stable up to 120 h (**Figures 1A,B**). Maximum growth was observed at 96 h (OD_660_ = 2.556) and decreased thereafter (**Figure 1C**). The pH decreased after 72 h and the medium became acidic (**Figure 1D**). In production medium containing ‘both glucose and corn oil’ as carbon sources, maximum reductions in ST and IFT were observed at 48 h and after 72 h reached to 28.56 ± 0.42 mN/m and 2.13 ± 0.09 mN/m from 53.69 ± 0.36 mN/m and 23.37 ± 0.41 mN/m respectively. The IFT was further reduced to 1.23 ± 0.02 mN/m after 96 h (**Figures 1A,B**). Maximum growth was observed at 72 h (OD_660_ = 3.372) and decreased thereafter (**Figure 1C**). The pH remained acidic throughout the experiments (**Figure 1D**).

In the production media containing both hydrophilic and lipophilic carbon sources (glucose and corn oil), reduction in ST and IFT were observed earlier as compared to production media containing either glucose or corn oil alone. At 48 h ST and IFT were reduced to 36.25 ±0.43 and 5.43 ±0.36 mN/m, respectively in medium containing ‘both glucose and corn oil.’ Whereas, in medium containing ‘either glucose’ or ‘only corn oil,’ STs and IFTs were reduced to ∼46.51 ±0.33 and ∼18.66 ±0.15 mN/m, respectively. Other researchers also reported that the highest production of SPLs was achieved when glucose was combined with a lipophilic carbon source (Zhou et al., 1992; Davila et al., 1994; Rau et al., 1996; Pekin et al., 2005). SPLs fermentation is a two-step process in which production occurs after growth when nitrogen source has been utilized (Cooper and Paddock, 1984; Davila et al., 1992). SPLs production has been described for cultures with only one carbon source, such as glucose (Hommel et al., 1994) or *n*-alkanes (Davila et al., 1994). However, production was significantly higher when two carbon sources (a carbohydrate based and lipidic one) were simultaneously provided. The various reported carbohydrate based substrates were glucose, sucrose (Klekner et al., 1991), lactose (Zhou and Kosaric, 1993), fructose or mannose (Göbbert et al., 1984). The reported lipidic substrates were *n*-alkanes (Davila et al., 1994), vegetable oils or waste frying oil (Zhou et al., 1992; Shah et al., 2007) or animal fats (Deshpande and Daniels, 1995). Continuous feeding of the lipid substrate, such as fatty acids (Rau et al., 1996) or fatty acid methyl or ethyl esters (Davila et al., 1992), has been shown to improve fermentation performances. The medium should also contain a source of nitrogen such as yeast extracts or corn steep liquor or additional nitrogen sources such as urea or ammonia, citrate buffering compounds and small amount of minerals such as Mg^2+^, Fe^3+^, Ca^2+^, Zn^2+^, and Na^+^ (Davila et al., 1992, 1997). We also observed quicker biosurfactant production in medium containing yeast extract and urea with both glucose and corn oil and therefore it was used for further studies.

### Stability Studies

**FIGURE 2:**
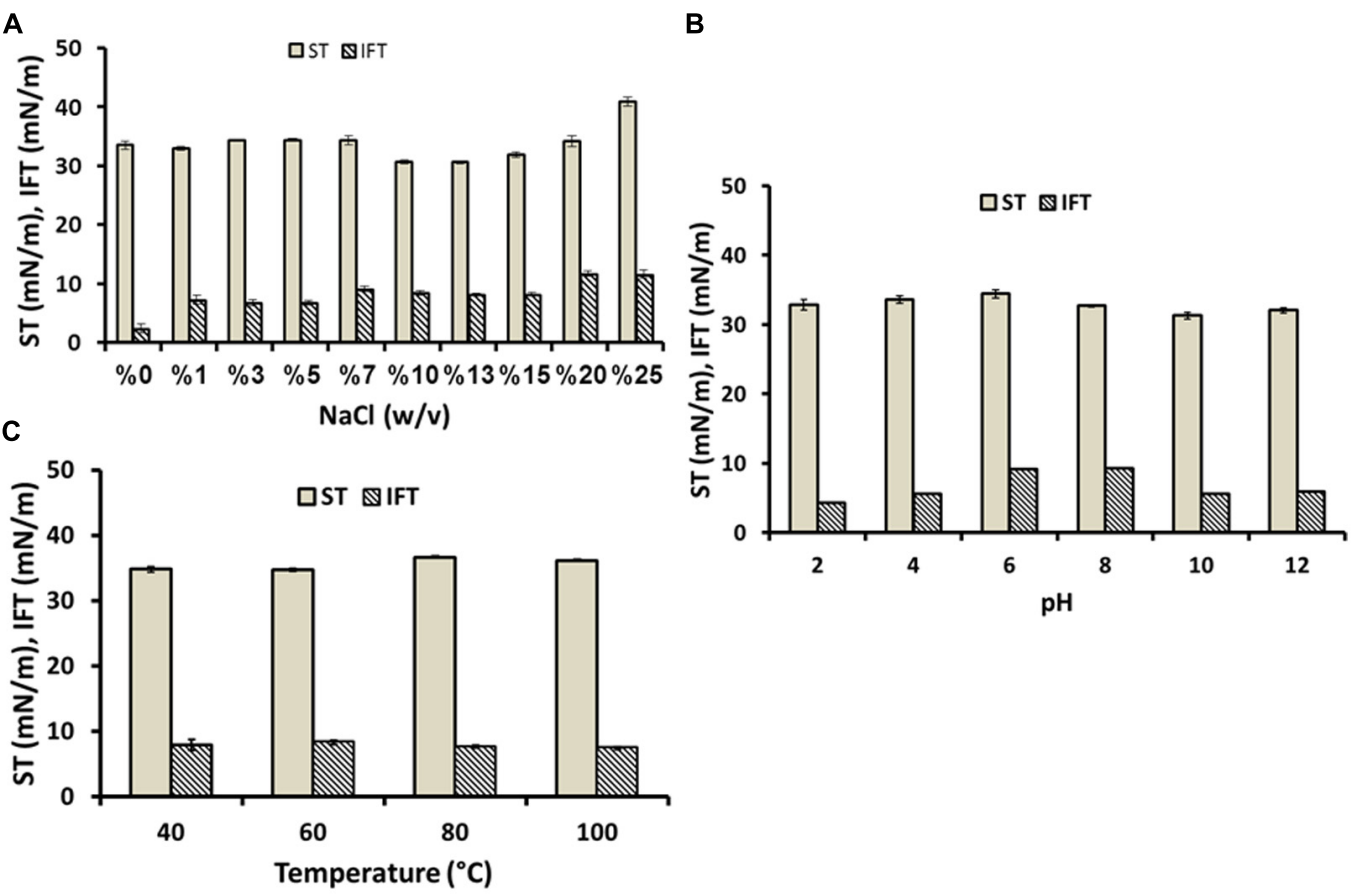
**Effect of salinity **(A)**, pH **(B)**, and temperatures **(C)** on the activity of biosurfactant produced by *C. bombicola* ATCC 22214**.

Temperature, pH and salinity are known to be one of the most important environmental factors influencing the performance of any component (including biosurfactants) to be used for enhanced oil recovery (EOR) purpose. For *ex situ* microbial enhanced oil recovery (MEOR) applications, biosurfactant must be stable at range of high temperature (≥50°C), effective at wide range of pH and salt concentration between 7 and 9% to ensure its applicability to induce oil recovery (Al-Sulaimani et al., 2011b). As shown in **Figure 2A**, the biosurfactant showed no changes in ST till 10–13% salt concentration, and at 15–25% concentration it increased from 31.93 to 40.90 mN/m. Whereas IFT increased to 7.28 mN/m after adding 1% salt and thereafter it remained stable till 15% salt concentration. Which showed clearly that even at high salt concentrations, biosurfactant can perform well and still retain some of its surface activity. As shown in **Figure 2B**, ST was quite stable at all pH range with values ranging between 31.0 and 34.0 mN/m. Whereas IFT was stable between pH < 6 and > 8, with corresponding value of 4.31–5.98 mN/m respectively, and at pH values between 6 and 8 it showed an increase in IFT value (**Figure 2B**). Thermal stability profile (**Figure 2C**) reveals that at different temperatures (40–100°C) the biosurfactant showed stability at all temperatures tested. These results indicated that the produced biosurfactant is suitable for MEOR applications as it was stable under extreme condition such as salinity, pH and temperature. This study showed that biosurfactants was quite stable at most salt concentration tested (13–15%), over a wide range of pH values tested (2–12), and at different temperatures (40–100°C). It is comparable with other types of biosurfactants reported by several researchers (Joshi et al., 2008, 2015; Ghojavand et al., 2008; Gudiña et al., 2010; Al-Sulaimani et al., 2011b).

### Emulsification Index (%E_24_)

**FIGURE 3:**
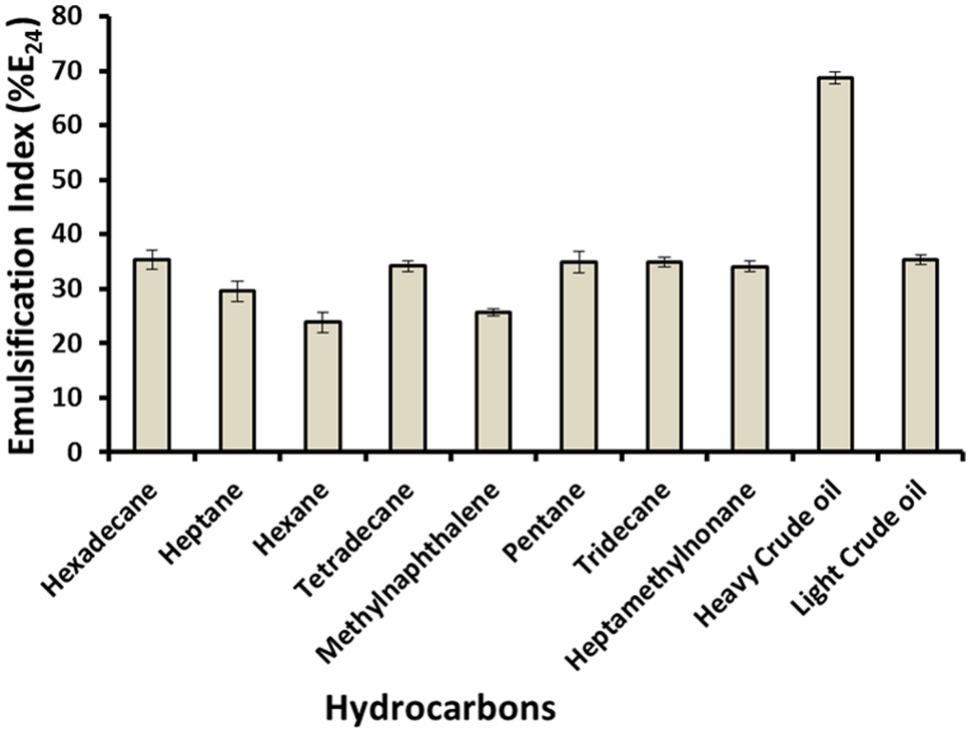
**Emulsification index of different hydrocarbons with biosurfactant produced by *C. bombicola* ATCC 22214**.

Emulsification is one of the features of biosurfactant which aids in enhancing the trapped oil from the oil wells. As shown in **Figure 3**, biosurfactant emulsified a variety of hydrocarbons. It gave high %E_24_ with heavy crude oil (68.75%), and with *n*-hexadecane, light crude oil, *n*-tetradecane, *n*-pentane, *n*-tridecane and 2,2,4,4,6,8-heptamethylnonane also formed stable emulsions, with %E_24_ between 34 and 35%. Whereas it showed lower %E_24_ (23.86–29.55%) against hexane, 1-methylnaphthalene and heptane. Higher %E_24_ with ‘heavy-crude oil’ can potentially help in heavy-oil recovery. Al-Wahaibi et al. (2014) reported lipopeptide type of biosurfactant produced by isolate *Bacillus subtilis* B30 in minimal medium or molasses medium, which showed around 15–55% emulsification of various hydrocarbons including light or heavy oils. Ismail and Dadrasnia (2015) also reported %E_24_ of novel *Bacillus* strain 139SI as 69% with crude oil, thus aiding in bioremediation of oil polluted water. We also observed similar results with biosurfactant produced by *C. bombicola*.

### Extraction and Characterization of Biosurfactant

The biosurfactant was extracted and purified by solvent extraction with ethyl acetate followed by washing with hexane which resulted in 2.42 g/l of crude product as yellowish brown powder (Supplementary Figure S1), after evaporation of hexane. In order to confirm the activity of the crude biosurfactant, ST and IFT were analyzed. The ST and IFT were found to be 30.32 ± 0.31 mN/m and 2.66 ± 0.38 mN/m respectively, which were quite similar to the values of ST and IFT of biosurfactant in broth, before extraction.

**FIGURE 4:**
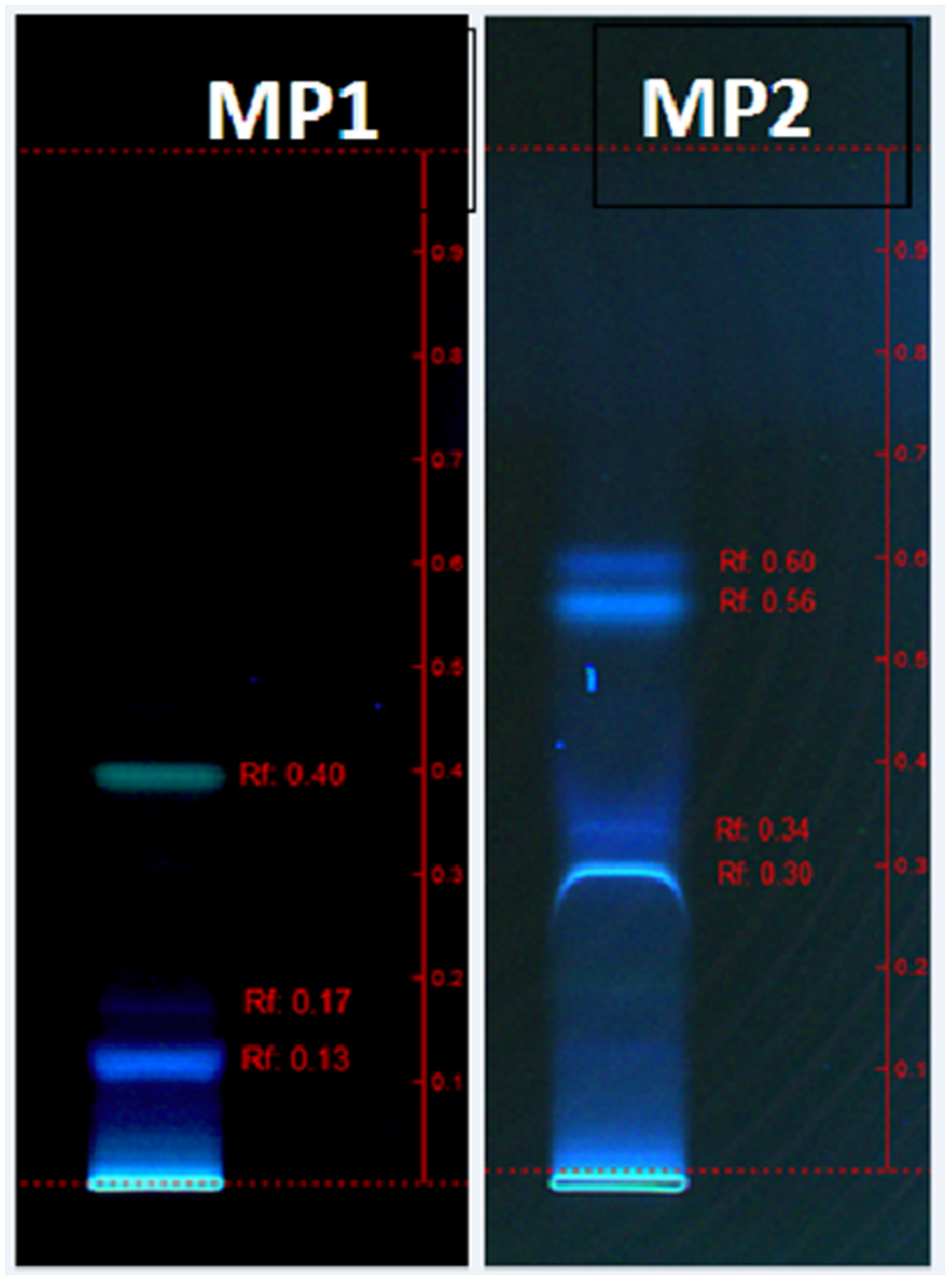
**Separation of biosurfactant by HPTLC in two different mobile phases MP1 and MP2**.

**FIGURE 5:**
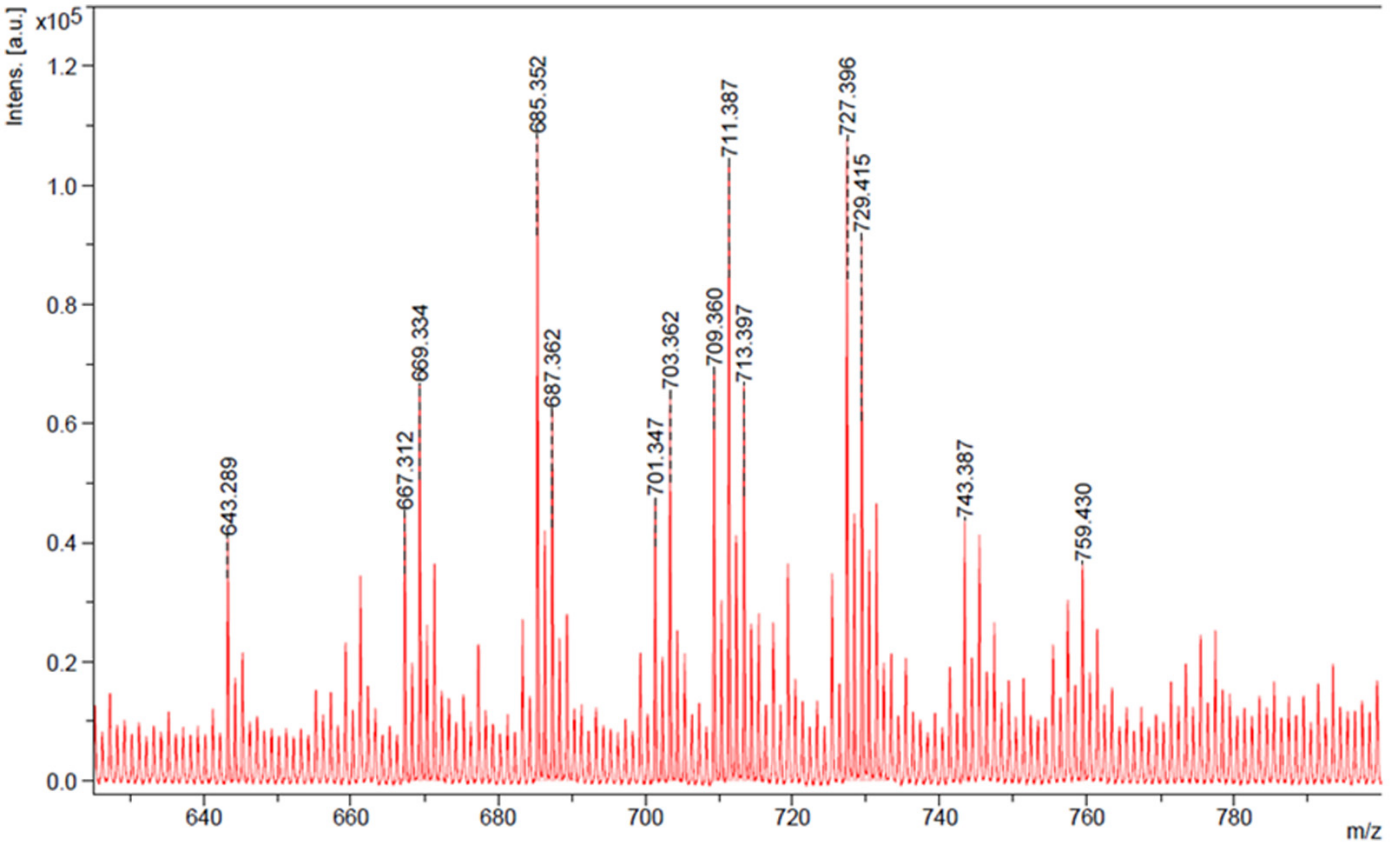
**Matrix-assisted laser desorption ionization – time of flight-mass spectroscopy (MALDI-TOF-MS) mass spectrum of produced biosurfactant**.

The characterization of the surface active components from the crude biosurfactant mixture is important to determine its molecular structure since their structural features may provide very useful information for future research purposes. The biosurfactant obtained using glucose-corn oil based production medium was identified and characterized by different analytical techniques. TLC or HPTLC has been reported as a useful technique for initial qualitative or quantitative analysis of biological active compounds (Al-Wahaibi et al., 2014). Thus we analyzed biosurfactant by HPTLC using two mobile phases, and the separated bands were directly extracted and analyzed for MS detection. Both the MP systems gave good separation of biosurfactant components and the visualization was better under UV 366 nm (**Figure 4**), and the extracted bands showed mass in the range of 300–795 (Supplementary Figure S2). The MALDI-TOF further confirmed the mass of the produced SPLs, of which major peaks were in the range of 643–759 Da (**Figure 5**). It is reported that typical structure of biosurfactant produced by *C. bombicola* – SPLs consists of a sophorose sugar β-glycosidically linked to terminal (or subterminally) hydroxylated fatty acid with chain lengths of 16–18, as a mixture of compounds. Where precursor fatty acids added to the production medium leads to formation of different forms of SPLs, which contains both acid and lactone SPLs, but lactones frequently represent the largest fraction of the product (Asmer et al., 1988; Davila et al., 1994; Joshi-Navare et al., 2013). We have used corn oil as a precursor, which is a mixture of fatty acids and thus multiple forms of SPLs with diverse hydrophobic moieties were expected. This was confirmed with the MALDI-TOF-MS analysis of the purified biosurfactant. The major peaks from the mass spectrum were correlated to sodium adducts [M^+^ + Na^+^] of different forms of SPLs, as derived from oleic acid (*m/z* 669, 687, 711, 729) were detected (**Figure 5**). Where major ions at *m*/*z* 711 and *m*/*z* 729 can be attributed to the [M^+^ + Na^+^] adduct ions for the lactone and free acid forms of the major diacetylated SPLs respectively (Asmer et al., 1988; Kurtzman et al., 2010). Other ions at *m*/*z* 669 and *m*/*z* 687 correspond to the monoacetylated forms of the major SPLs, whereas the non-acetylated forms were not observed in current biosurfactant. The observed difference between these two sets of ions (729–711 and 687–669) can be attributed to the mass difference between the free acid form and the ester-linked lactone form of SPLs respectively. Thus the mass of produced SPLs can be calculated as from ∼646 to 706. Kurtzman et al. (2010) reported production and characterization of SPLs by multiple species of the *Starmerella* (*Candida*) *bombicola* yeast clade, using high-throughput MALDI-TOF-MS analysis. They reported SPLs production using oleic acid as fatty acid precursor along with other components in the production medium, and thus observed only oleic acid containing SPLs. Whereas we have used corn oil as fatty acid precursor and observed other minor SPLs derived from other types of acids present in corn oil, as sodium adducts [M^+^ + Na^+^] derived from palmitic (*m/z* 685, 703), linoleic (*m/z* 709, 727), and trace amounts of stearic (*m/z* 713) and arachidic (*m/z* 759) acid (**Figure 5**). The mass difference (18 Da) between the two sets of ions derived from palmitic and linoleic acids are again indicative of the free acid and lactone forms of the minor SPLs respectively. Similar type of SPLs ions were identified and reported previously from *C. bombicola* and other yeast species using fast atom bombardment MS or MALDI-TOF MS (Asmer et al., 1988; Kurtzman et al., 2010; Joshi-Navare et al., 2013).

**FIGURE 6:**
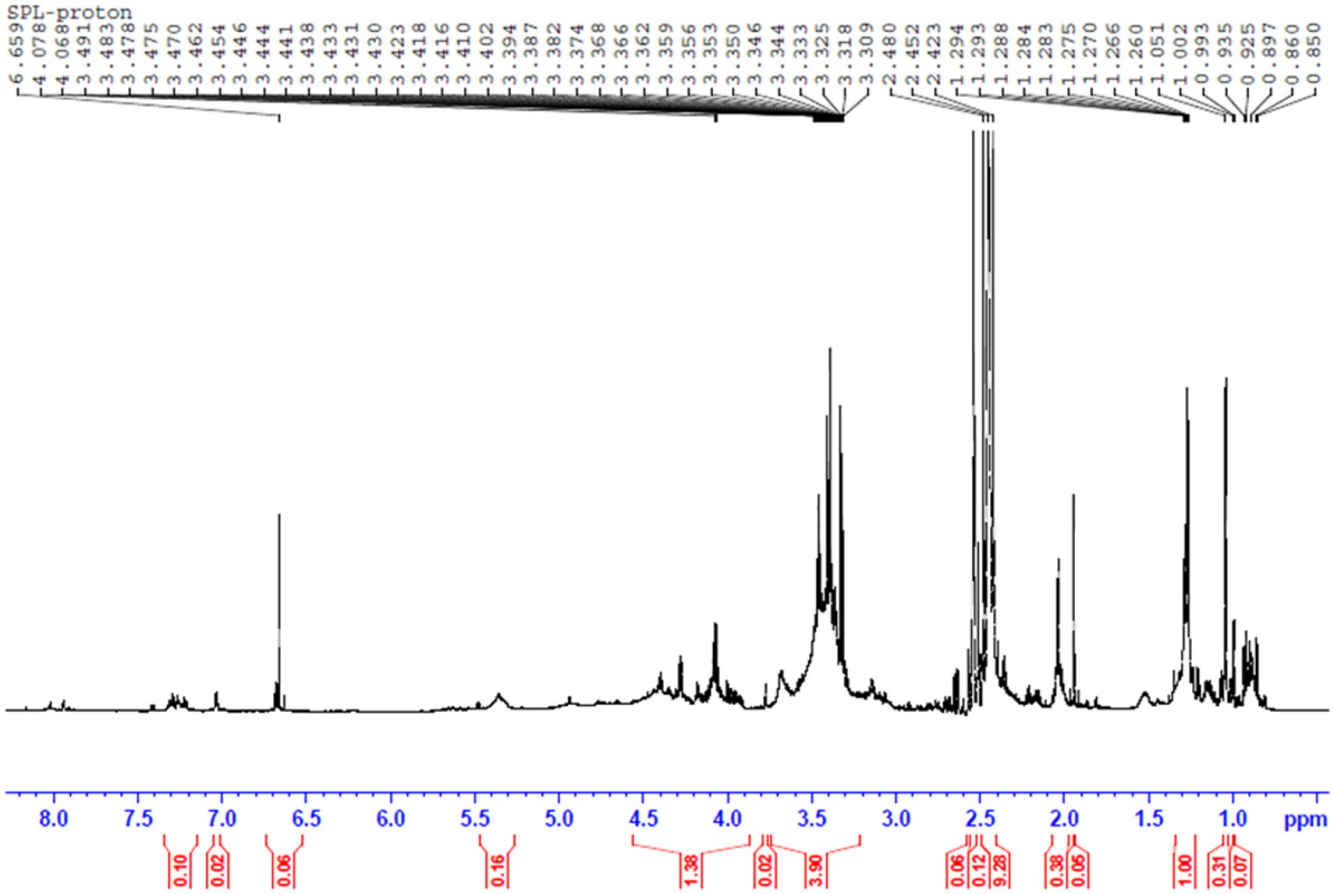
**The proton (^1^H) NMR spectrum of produced biosurfactant**.

**FIGURE 7:**
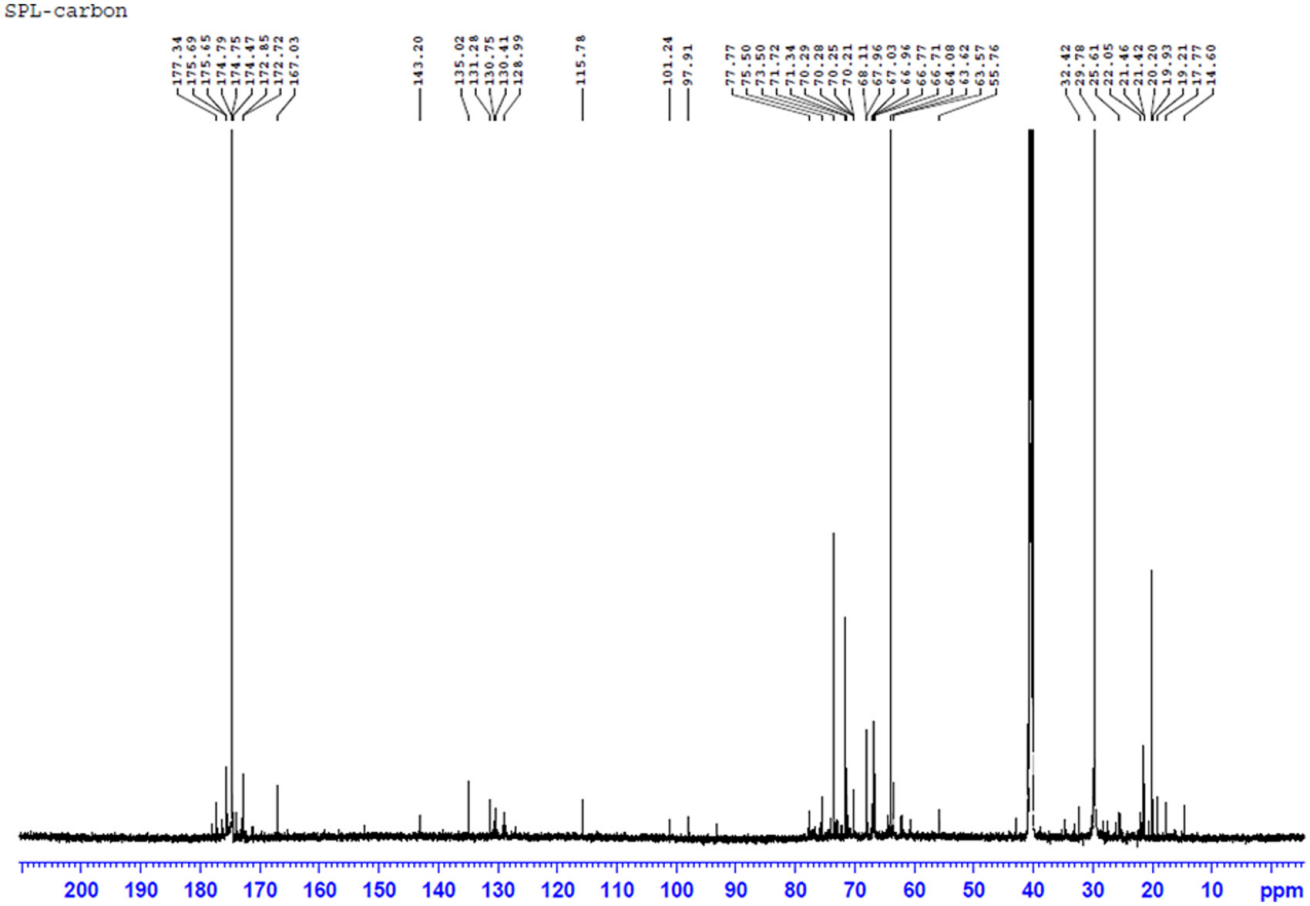
**The proton decoupled-carbon (^13^C) NMR spectrum of produced biosurfactant**.

The structures of biosurfactants were further confirmed by ^1^H, and proton decoupled ^13^C – NMR and the results are shown in **Figures 6** and **7**. The ^1^H NMR spectrum of the purified biosurfactant in CDCl_3_ was assigned to a typical glycolipid-type structure and characteristic proton chemical shift peaks could be observed (**Figure 6**). Multiple signals of protons of (-CH_3_) below 1.0 ppm and of (-CH_2_) between 1.0 and 1.4 ppm revealed the existence of a linear alkane, and signals at 5.3–5.4 ppm revealed presence of (-CH = CH-) group in the fatty acid chain. The signal at 5.3–5.4 ppm is attributable to an unsaturated hydrocarbon moiety, also consistent with the oleic acid derived SPLs signatures in MALDI-TOF-MS analysis. Protons of (-CH_2_) bonded to carboxylic group of fatty acid resonated at ∼1.99 ppm, and ∼2.09 ppm revealed the presence of (-COCH_3_) group in biosurfactant. Resonance of protons belonging to sophorose moiety resulted in peaks within the region 4–4.5 ppm, and the other protons of sugar were resonated at 3.18–3.8 ppm.

The ^13^C NMR spectrum of the biosurfactant showed the presence of two (=CH-) groups in the fatty acid chain moiety corresponding to signals at 130.41 ppm and 128.99 ppm; and few more (=CH-) groups in the fatty chain moiety were resonated between 128 and 130 ppm, which may be probably due to contribution from other interfering group of biosurfactant in the sample (**Figure 7**). In addition, several (-CH_2_-) groups in the fatty chain moiety were also resonated at 20∼35 ppm. The spectrum also revealed signals of glucose- C-1″ at 101.24 ppm, glucose-C-6′, and glucose-C-6″ at 64.08, 62.29 ppm; the other carbon atoms of glucose were resonated between 70∼78 ppm. The peaks derived from the carbonyl groups (-CO-) were shown at 170∼177 ppm. The chemicals shifts were comparable to previous reports (Daverey and Pakshirajan, 2009, 2010; Wadekar et al., 2012; Joshi-Navare et al., 2013). The MALDI-TOF-MS and NMR results further confirmed the biosurfactant as a mixture of SPLs with mono and diacetylated SL as mixture of different fatty acids in acidic or lactonic form.

### Core-flood Experiments

**FIGURE 8:**
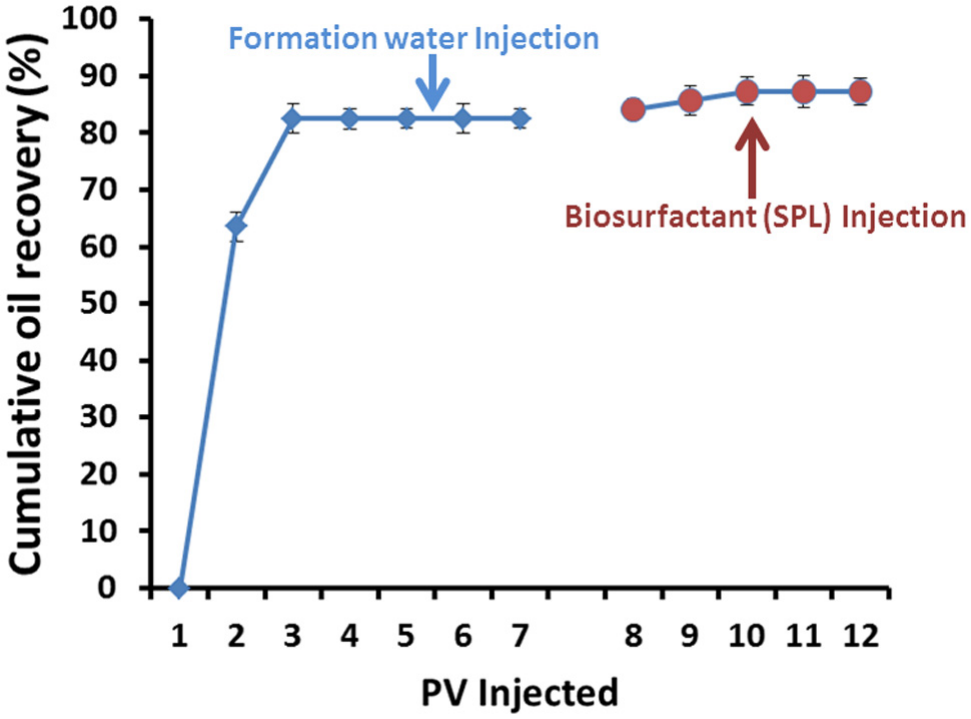
**Cumulative oil recovery as a function of pore volumes (PV) injected of formation water and biosurfactant-SPLs**.

An area of considerable potential for biosurfactant application is in the field of MEOR. Since oil is a critical energy source that drives industrialization and sustained economic development of the world (Youssef et al., 2006). McInerney et al. (2005) documented that current oil production technologies recover only about one-third to one-half of the oil originally present in an oil reservoir. So, EOR methods were developed to recover oil remaining in reservoirs after primary and secondary recovery procedures. MEOR is an important tertiary recovery technology which utilizes microorganisms and/or their metabolites for residual oil recovery (Banat, 1995) which was attempted more than 60 years ago (Hitzman, 1991). We checked the potential of produced SPLs in MEOR using core-flooding experiments. The PV of the core-sample was 18 cm^3^. Initial oil saturation (So_i_) was calculated to be 55–60% after oil flooding. It was found that after injecting 5–8 PV of formation water, no more oil was produced and residual oil saturation (So_r_) was about 20%. Extra oil recovery was observed after injecting 4–5 PV SPLs, where 27.27% of So_r_ was produced (**Figure 8**). An increase in oil recovery in *ex situ* MEOR experiments was detected following SPLs injection. This is similar to reports shown by other researchers for different types of biosurfactants (Al-Sulaimani et al., 2011b; Castorena-Cortés et al., 2012; Joshi et al., 2015). No study was reported before to test the ability of SPLs in enhancing oil recovery using core flood experiments. To the best of our knowledge, this study was the first to explore the efficiency of SPLs to recover oil and it can be used in biosurfactant based MEOR applications. There are various experiment at laboratory scale that have been used to prove the effectiveness of using biosurfactants for microbial enhancing oil recovery, such experiments include sand-pack columns or core-flooding and field trials (Yakimov et al., 1997; Youssef et al., 2006; Joshi and Desai, 2013; Al-Wahaibi et al., 2014). This research is the first report to investigate the ability of SPLs produced by *C. bombicola* ATCC22214 to enhance oil recovery using core-flooding. Biosurfactants like SPLs are considered as green alternatives for chemical counterparts, as it can be produced from renewable sustainable resources. Production economy is still considered as bottleneck for widespread biosurfactants applications, which hinders the field-scale applications. The cost of production can be drastically reduced when agro-industrial waste products (like molasses) or used waste frying oil (as fatty acid supplement) be used as raw material for SPLs production at large scale fermentation (Makkar et al., 2011; Banat et al., 2014). Once scaled-up, these SPLs can be used for biosurfactant based MEOR or environmental bioremediation, without much purification so that the cost of application can be further reduced. Thus large scale production of SPLs using cheaper raw materials and field-scale MEOR application are further proposed.

## Conclusion

This study shows that corn oil could be used as a carbon source along with glucose to produce SPLs by the yeast *C. bombicola* ATCC22214. The SPLs showed reduction of ST and IFT; high %E_24_ against various hydrocarbons including light and heavy crude oils, and also showed high stability under extreme conditions of salinity, pH and temperature. It was characterized as a mixture of SPLs, using different analytical techniques. To the best of our knowledge this is the first report on using SPLs for testing MEOR applications. Core-flooding studies revealed that it can recover 27.27% of residual oil (S_or_) trapped between the pores of Berea sandstone cores, which highlight the potential applications of SPLs in MEOR.

## Conflict of Interest Statement

The authors declare that the research was conducted in the absence of any commercial or financial relationships that could be construed as a potential conflict of interest.
