# Supplementary material for: Sophorolipids Production by Candida bombicola ATCC 22214 and its Potential Application in Microbial Enhanced Oil Recovery
Source: Front Microbiol. 2015 Nov 26;6:1324. doi: 10.3389/fmicb.2015.01324 (PMC4659913; doi:10.3389/fmicb.2015.01324)
Supplement: Supplementary file 1 [file Image_1.PDF]

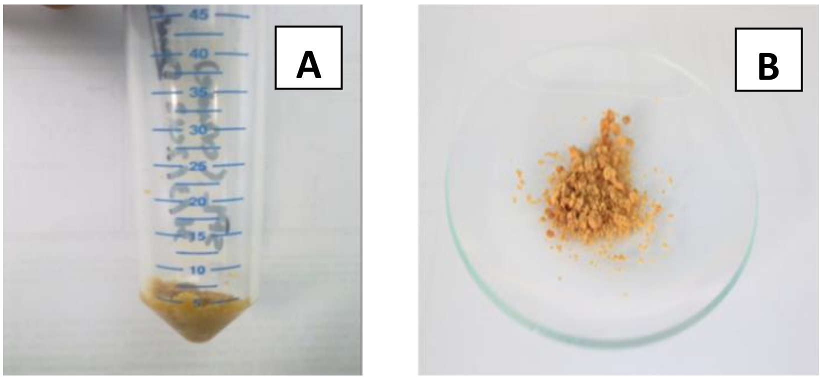

Figure S1: The extracted crude SPLs before (A) and after (B) evaporation of hexane.

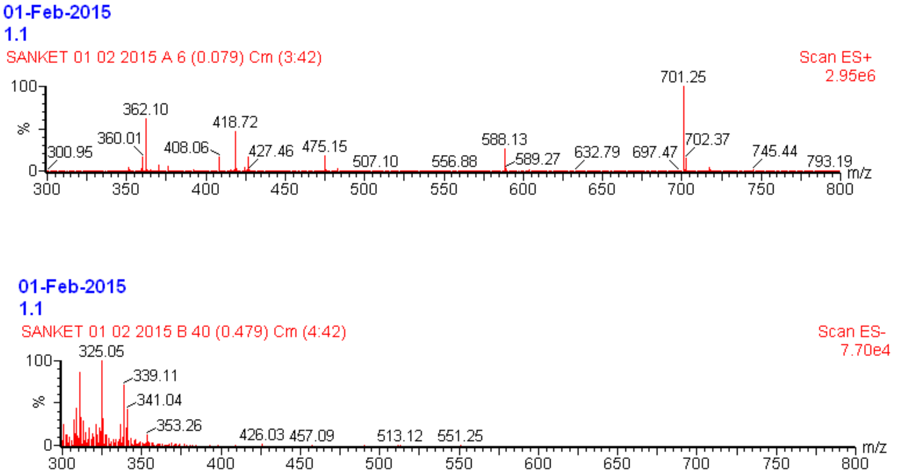

A

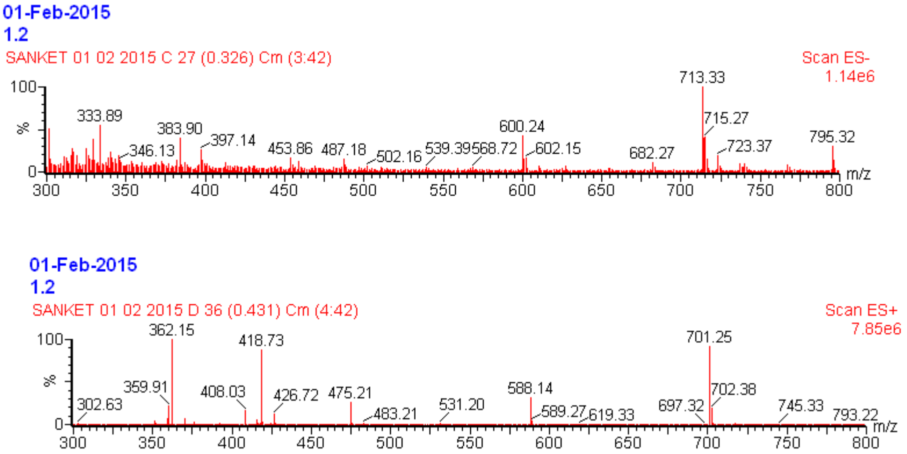

B

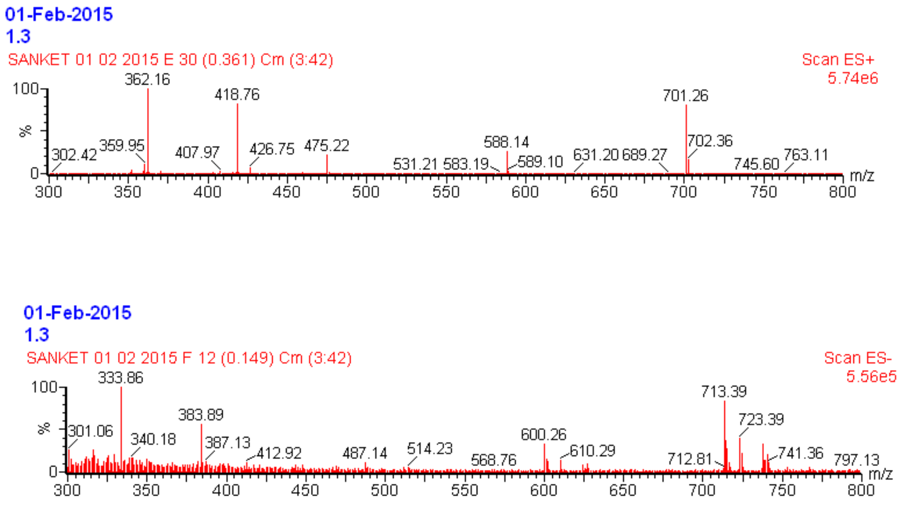
01-Feb-2015
1.3
SANKET 01 02 2015 E 30 (0.361) Cm (3:42)
Scan ES+
5.74e6
362.16
418.76
701.26
302.42
359.95
407.97
426.75
475.22
531.21
583.19
588.14
589.10
631.20
689.27
702.36
745.60
763.11
m/z
01-Feb-2015
1.3
SANKET 01 02 2015 F 12 (0.149) Cm (3:42)
Scan ES-
5.56e5
333.86
301.06
340.18
383.89
387.13
412.92
487.14
514.23
568.76
600.26
610.29
712.81
713.39
723.39
741.36
797.13
m/z

C

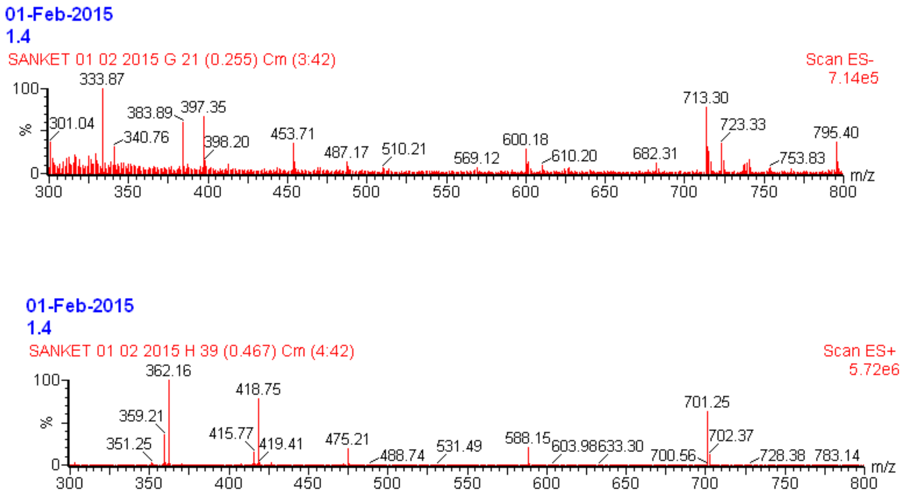
01-Feb-2015
1.4
SANKET 01 02 2015 G 21 (0.255) Cm (3:42)
Scan ES-
7.14e5
333.87
301.04
340.76
383.89
397.35
398.20
453.71
487.17
510.21
569.12
600.18
610.20
682.31
713.30
723.33
753.83
795.40
m/z
01-Feb-2015
1.4
SANKET 01 02 2015 H 39 (0.467) Cm (4:42)
Scan ES+
5.72e6
362.16
418.75
359.21
351.25
415.77
419.41
475.21
488.74
531.49
588.15
603.98
633.30
700.56
701.25
702.37
728.38
783.14
m/z

D

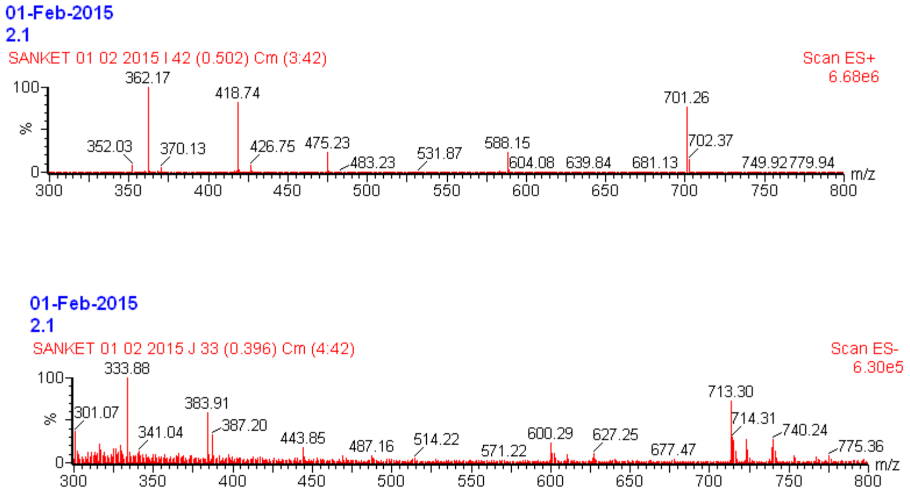
01-Feb-2015
2.1
SANKET 01 02 2015 I 42 (0.502) Cm (3:42)
Scan ES+
6.68e6
362.17
418.74
352.03
370.13
426.75
475.23
483.23
531.87
588.15
604.08
639.84
681.13
701.26
702.37
749.92
779.94
m/z
01-Feb-2015
2.1
SANKET 01 02 2015 J 33 (0.396) Cm (4:42)
Scan ES-
6.30e5
333.88
301.07
341.04
383.91
387.20
443.85
487.16
514.22
571.22
600.29
627.25
677.47
713.30
714.31
740.24
775.36
m/z

E

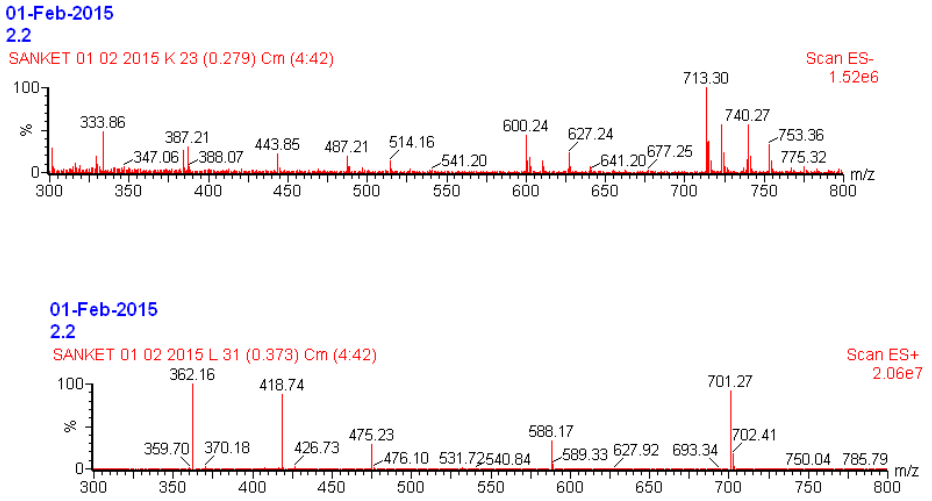

F

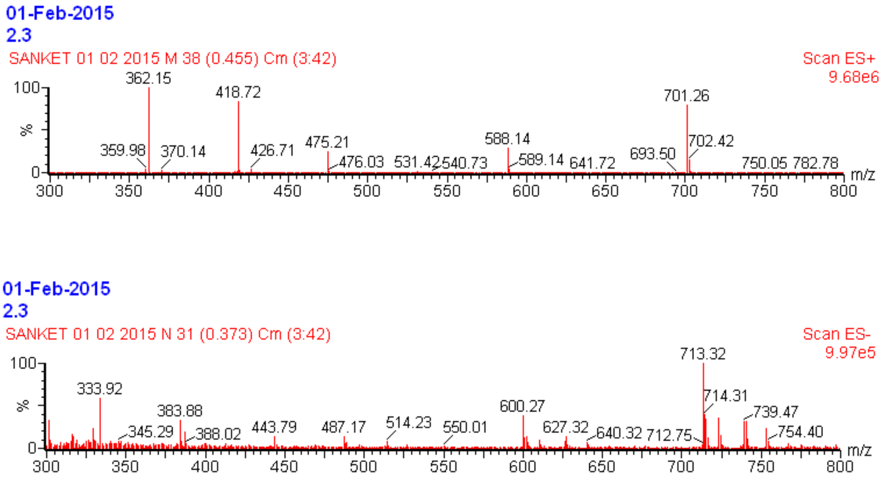

G

Figure S2: The mass determination of separated bands of biosurfactants by HPTLC-MS (A-G).
